# Should We Build “Obese” or “Lean” Anaerobic Digesters?

**DOI:** 10.1371/journal.pone.0097252

**Published:** 2014-05-15

**Authors:** Aurelio Briones, Erik Coats, Cynthia Brinkman

**Affiliations:** 1 Department of Plant, Soil and Entomological Sciences, University of Idaho, Moscow, Idaho, United States of America; 2 Department of Civil Engineering, University of Idaho, Moscow, Idaho, United States of America; University Paris South, France

## Abstract

Conventional anaerobic digesters (ADs) treating dairy manure are fed with raw or fermented manure rich in volatile fatty acids (VFAs). In contrast, pre-fermented AD (PF-AD) is fed with the more recalcitrant, fiber-rich fraction of manure that has been pre-fermented and depleted of VFAs. Thus, the substrate of PF-AD may be likened to a lean diet rich in fibers while the pre-fermentation stage fermenter is fed a relatively rich diet containing labile organic substances. Previous results have shown that conventional and pre-fermented ADs fed with raw or pre-fermented manure, respectively, produced comparable methane yields. The primary objective of this study was to characterize, using next-generation DNA sequencing, the bacterial communities in various bioreactors (pre-fermentation stage fermenter; various operational arrangements PF-AD; conventional single-stage AD; and a full scale AD) and compare the *Firmicutes* to *Bacteroidetes* (F/B) ratios in these different systems. *Firmicutes* and *Bacteroidetes* constituted the two most abundant phyla in all AD samples analyzed, as well as most of the samples analyzed in the fermenters and manure samples. Higher relative abundance of *Bacteroidetes*, ranging from 26% to 51% of bacteria, tended to be associated with PF-AD samples, while the highest relative abundance of *Firmicutes* occurred in the fermenter (maximum of 76% of bacteria) and manure (maximum of 66% of bacteria) samples. On average, primary stage fermenters exhibited microbiological traits linked to obesity: higher F/B ratios and a ‘diet’ that is less fibrous and more labile compared to that fed to PF-AD. On the other hand, microbial characteristics associated with leanness (lower F/B ratios combined with fibrous substrate) were associated with PF-AD. We propose that bacterial communities in AD shift depending on the quality of substrate, which ultimately results in maintaining VFA yields in PF-AD, similar to the role of bacterial communities and a high fiber diet in lean mice.

## Introduction

Obesity and or leanness are not traits commonly associated with engineered systems like anaerobic digesters (ADs). However, AD microbial consortia may be greatly influenced by substrate qualities that in the gut, elicit metabolic responses that lead to either obesity or leanness. Like gut bacteria, ADs rely on the activities of different functional groups of anaerobic microbes that work together to degrade organic matter to produce methane. Thus, ADs and many animal digestive systems are faced with similar metabolic challenges that require similar biochemical reactions involving hydrolysis, acidogenesis, acetogenesis, and methanogenesis. The first three processes ultimately lead to the production of volatile fatty acids (VFAs) that are critical intermediates in both animal and engineered systems. The fate of VFAs bifurcate depending on the system: they are mainly absorbed and converted to energy and/or body mass in animals or converted to methane in ADs. Most of the methane formed in animals is considered energetically wasteful, although hydrogen consumption by methanogens may enhance fermentation. Regardless of the system however, bacteria play similar roles in the generation of VFAs from organic matter.

Our interest in anaerobic digestion – and ultimately to the potential linkage between obesity/leanness and substrate – stems from the need to address the environmental impacts posed by manure produced from intensive dairy operations. Since ADs produce energy while treating an organic source of pollution such as excessive manure, anaerobic digestion of dairy waste appears to be a win-win situation. However, it has not gained traction in places like the United States because of unfavorable economics driven primarily by low electricity and natural gas rates [1]. One way to address the low adoption of ADs in the US and other places is to diversify and create added value to the bioproducts from ADs. Thus we have proposed that anaerobic digestion be conducted as a two-stage operation wherein the first stage operates as a high-rate fermenter to produce VFAs from the labile fraction of dairy manure [1]. VFAs are diverted to other bioreactors to produce high value bioproducts such as bioplastics and biofuels [1]–[3]. The thickened, pre-fermented manure is fed to the AD for biogas production. In contrast to conventional single-stage or second-stage AD that is fed with raw manure and VFA-rich supernatant from a primary fermenter respectively, our pre-fermented AD (PF-AD) is fed with the more recalcitrant, fiber-rich fraction of manure that persists through the pre-fermentation stage. Thus, the PF-AD substrate may be likened to a lean diet rich in fibers while the primary stage fermenter is fed a relatively rich diet containing labile organic substances that are more easily converted to VFAs. A single-stage AD (SS-AD) supports both the fermentation of raw manure and subsequent anaerobic digestion and receives the same feed type as a primary fermenter. However, the microbial community of a SS-AD will reflect characteristics of anaerobic digestion, since conditions such as turnover time of solids (solids retention time  =  SRT) are imposed to optimize methanogenesis.

Recent findings relating gut microbiota to the obese and lean states suggest that leanness is a characteristic associated with the interaction between increased abundance of *Bacteroidetes* relative to *Firmicutes* (the two most dominant phyla in the gut) and the presence of high fiber (and low fat) in the diet [4]. This combination produces high levels of VFAs derived from plant fiber that promotes leanness by inhibiting fat accumulation in adipose tissue and through other possible physiological mechanisms [5]–[7]. If similar bacterial processes were to occur in the PF-AD, then this may help explain the high rates of methane production that are routinely achieved from pre-fermented manure when compared to single-stage AD fed with raw manure [8]. This also suggests that the microbiota in ADs fed with a high-fiber ‘diet’ adjusts to the substrate and thus remains potentially productive in terms of VFA production as has been observed in mice fed with high fiber diets.

The primary objective of this study was to characterize the bacterial communities in various bioreactors (primary stage fermenter; various operational arrangements PF-AD; and conventional single-stage AD) and compare the *Firmicutes* to *Bacteroidetes* ratios in these different systems. To obtain a more complete picture of the microbiology of AD, we performed similar analysis on samples obtained from a full scale AD processing manure from 11,000 dairy cows. Our second objective was to identify the key bacterial populations that are associated with fermentation and anaerobic digestion. Identifying the average bacterial composition and core microbiomes (a core microbiome is comprised of members common to two or more microbial assemblages associated with a habitat [9]) in each process are keys to better understand the links between substrate quality, microbiology and bioreactor performance. This may also be the first step in developing useful mixtures of bacteria for bioaugmentation during times of reactor failure or other perturbations.

## Materials and Methods

### Description of the Bioreactors

Concurrent with the research in the present study, we have been investigating multiple issues related to PF-AD operation and optimization [1], [8]. Samples from these earlier studies were included in the present study for next-generation DNA sequencing (NGS) analysis. Construction of the laboratory-scale SS-AD and PF-AD digesters (Table 1), with the exception of the 0.4L PF-AD, is described elsewhere [8]. The 0.4L PF-AD consisted of a 0.5L glass incubation bottle, capped with a rubber stopper (vented to a wet tip gas flow meters (wettipgasmeter.com), placed inside a water bath and mixed using a magnetic stir plate. Digesters SS-AD and all PF-ADs were operated as completely and continuously mixed systems under the conditions specified; each AD was manually decanted and fed once daily to maintain the SRT/HRT (solids/hydraulic retention time). Each AD received manure obtained from the University of Idaho dairy (Moscow, ID, USA), a facility that maintains around 100 milking cows. The manure substrate was supplied to the ADs either as raw and unprocessed manure, or pre-fermented in a laboratory-scale fermenter (see Coats et al. 2012 [8] and Coats et al. 2013 [1] for additional details). Moreover, the fermented solids were recovered either through a fine-mesh screen or through centrifugation (10,000 rpm for 10 minutes). ADs fed with all pre-fermented solids (*via* centrifugation), only the coarse solids (*via* screening), and only the residual fine solids (*via* centrifugation of screened effluent) were separately investigated.

**Table 1:** Summary of operational and performance characteristics of anaerobic digesters and fermenters that were sampled for next generation sequencing.

	Single-stage AD (SS-AD)	Pre-fermented AD PF-AD	PF-AD	PF-AD	PF-AD	PF-AD	PF-AD	Large Scale AD (LS-AD)	Fermenter
**No. of samples sequenced (labeling codes)**	6 (O1-O6)	6 (T1-T6)	1 (T7)	2 (T8, T9)	1 (T10)	2 (T11, T12)	1 (T13)	4 (D1-D4)	5 (F1-F5)
**Received Pre-fermented Manure?**	No	Yes	Yes	Yes	Yes	Yes	Yes	No	No
**Substrate Characteristics**	Raw manure	All solids from manure fermenter	All solids from manure fermenter	Screened solids (large fraction) from manure fermenter	All solids from manure fermenter	Screened solids (large fraction) from manure fermenter	Centrifuged solids (finer fraction) from screened fermenter effluent	Manure from 11,000 cows	Raw manure
**Operating Volume, L**	40	40	40	40	40	40	0.4	3.78×10^6^	20
**SRT = HRT, d**	20	16	20	20	30	30	20	n.d.*	4
**Organic Loading Rate, gVS/L-d**	3.7	4.2	3.31	3.39	2.60	3.14	3.66	n.d.	8.75–9.5
**Operating Temperature**	35°C	35°C	35°C	35°C	35°C	35°C	35°C	37–38°C	35°C
**Average Methane Yield (L CH4/L d**	0.70	0.71	0.56	0.59	0.55	0.59	0.48	n.d.	n.d.

*n.d.  =  not determined.

### DNA Extraction and PCR

Genomic DNA was extracted from biomass obtained from each AD using the MO BIO PowerSoil DNA Isolation Kit (MO BIO Laboratories, Inc., Carlsbad, CA, USA). Biomass samples were collected on nine dates during AD operational analysis period. Samples were stored at −20°C until further use. Amplification of 16S rRNA fragments for next-generation (Ion Torrent) DNA sequencing was carried out on genomic DNA using *Bacteria*-specific primer set 338F (5′-ACT CCT ACG GGA GGC AGC AG-3′) and 533R (5′- TTA CCG CGG CTG CTG GCA C-3′) [10]. The PCR reaction was performed using 50 ng of DNA template with 5 minutes of initial denaturation at 94°C and 20 cycles of 94°C for 30 seconds, 56°C annealing for 30 seconds, 72°C extension for 1 minute, and a 72°C final extension for 7 minutes. DNA was purified using GeneJet Gel Extraction Kit (Thermo Scientific, Pittsburgh, PA, USA) and quantified using Synergy H1 micro plate reader (BioTek, Winooski, VT, USA).

### Ion Torrent Sequencing and Data Analysis

End-repair, adapter ligation and nick repair of 16S rRNA gene amplicon libraries were done using Ion Plus Fragment Library Kit (Life Technologies Corp., Carlsbad, CA, USA; Cat # 4471252) according to the manufacturer's instructions. Each sample library was amplified and then purified using Agencourt AMPure XP system (Beckman Coulter Inc., Brea, CA, USA). Libraries were quantified using an Agilent Bioanalyzer (Agilent Technologies Inc., Santa Clara, CA, USA). Template preparation and enrichment on Ion Sphere Particles was done using One Touch 200 Template Kit version 2 (Cat # 4478320) on an Ion One Touch Enrichment System. Sequencing was done with the Ion PGM 200 Sequencing Kit (Cat # 4474007) using an Ion Torrent Personal Genome Machine at the Molecular Research Core Facility in Idaho State University (Pocatello, Idaho, USA).

Data processing was done using Mothur software [11] designed to process and analyze 16S rRNA gene sequence data, which was implemented within the Galaxy bioinformatics platform [12]. Sequence reads of less than 150 bp were deleted from the data sets as were sequences with average base quality score of less than 24 (a quality score of 20 corresponds to 99% base call accuracy). Chimeric sequences were removed using the UCHIME algorithm [13] and further denoised (i.e., removal of sequences that most likely arise from sequencing errors) using a pseudo-single linkage algorithm implemented in Mothur that is based on a method described by Huse et. al., 2010 [14].

Analysis of alpha diversity and richness (i.e., diversity/richness within samples), classification of sequences and rarefaction analysis were done for each sample library using a down-sampled library size of 96,000 sequences to prevent possible bias due to effects of variable library sizes. Determinations of diversity, richness and classifications were done on operational taxonomic units (OTUs) defined by clustering of sequences at 3%, 5% and 20% levels of dissimilarity, nominally corresponding to groupings at the species, genus and phylum levels. Analysis of beta diversity (compositional similarity among samples) was performed on pooled sequences representing random sub-samples of 9,600 sequences from 21 AD samples (Table 2), three manure samples and a compost sample included as an outgroup. Compositional similarity among samples was determined using principal coordinates analysis (PCoA) implemented in Mothur. The same data set used for PCoA was used to compare and determine statistical significance of differences between bacterial communities of samples using AMOVA (analysis of molecular variance) as implemented in Mothur. The core microbiomes of two major groupings were determined from the beta-diversity data set; these consisted of a group containing all of the manure and fermenter samples (FERMAN, consisting of four fermenter and 3 manure sequence libraries) and a group containing all of the AD samples (ALL-AD, consisting of 17 AD libraries; see Table 2 for details). The core microbiomes of these two major groups were determined by identifying the sequences and OTUs that are shared among all the members of each major group. The average microbiomes of each group were determined by identifying the sequences and OTUs of a random sub-sample of sequences equal in size to the respective core microbiomes of each major group, i.e., FERMAN and ALL-AD. The sizes of the core and average microbiomes of FERMAN and ALL-AD were 24,880 and 30,849 sequences, respectively.

**Table 2:** Sampling dates of samples used for next-generation DNA sequencing and subsequent beta-diversity analysis.

Sampling date	Bioreactor type	Number of samples sequenced	Samples included in beta-diversity analysis
28-Jan-11	SS-AD	2	O1
	PF-AD	2	T2
25-Feb-11	FERMAN	2	F1, M1
	SS-AD	1	O3
	PF-AD	1	T3
6-Apr-11	SS-AD	2	O4
	PF-AD	2	T4
20-Apr-11	FERMAN	2	F2, M2
	SS-AD	1	O6
	PF-AD	1	T6
10-Oct-11	FERMAN	3	F3, F4, M3
	PF-AD	2	T7, T10
9-Feb-12	PF-AD	2	T8, T11
18-Jun-12	PF-AD	3	T9, T12, T13
9-Aug-12	LS-AD	4	D1, D2
15-Jan-13	FERMAN	1	

FERMAN  =  fermenters and manure.

SS-AD  =  single-stage anaerobic digester.

PF-AD  =  pre-fermented anaerobic digester.

LS-AD  =  large-scale anaerobic digester.

### Sequence accession numbers

Sequence data were deposited at the National Center for Biotechnology Information Sequence Read Archive (accession SRP035673).

## Results

We processed a total of 28 bioreactor (Table 1) and 3 manure samples for Ion Torrent sequencing. The average sequence length after removal of chimeras and non-bacterial sequences was 181.8 nucleotides and the average sequence quality (Phred score) was 26.4, meaning that the average probability of an incorrect base call is 1 in 518.5, or the average base call accuracy was 99.7%.

### Alpha Diversity Analysis

Estimates of OTU richness in all the bioreactors and manure were based on a target sample size of 96,000 sequences per sample, which in all cases provided greater than 90% coverage at the 5% dissimilarity level (nominally corresponding to grouping by genus). At both 3% and 5% (nominally species and genus) dissimilarity, the highest average numbers of OTUs observed were found in the large-scale anaerobic digesters processing manure from thousands of cows (Table S1). Rarefaction curves of the most diverse (LS-AD) and least diverse (manure) data sets suggest that these have been sampled sufficiently (Figure S1). A surprising result is the high levels of OTU diversity that were observed at the phylum level in the fermenter samples (Table S1). Even with the large sample sizes used in this study, OTUs occurring once or twice (singletons or doubletons) may constitute a dominant fraction of the total OTUs. The Chao1 estimator of OTU richness takes into account the frequency of singletons and doubletons in estimating OTU richness. When comparing the different sample types by Chao1 richness, the differences observed in OTU richness were even more apparent (Table S1), suggesting that much of the diversity in LS-AD lies in the rare phylotypes. Similarly, much of the diversity at the phylum level in the fermenters are driven by rare phylotypes detected as singletons.

### Beta diversity analysis

**Figure 1:**
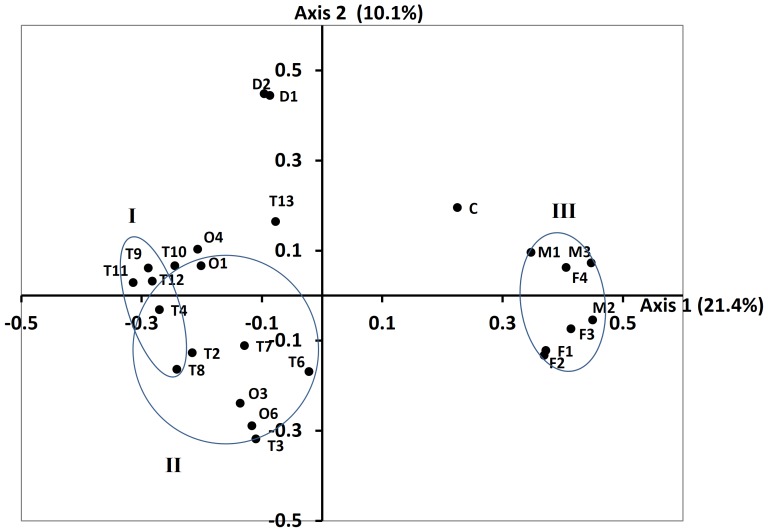
Principal coordinates analysis showing the compositional similarities among the bacterial communities of manure, fermenter, and AD samples as well as a compost sample (C) included as an outgroup. The 21 fermenter and AD samples included in the analysis and the sample codes are listed in Table 2; all samples represent a total of 240,000 sequences. Coordinates were derived from a Bray-Curtis distance matrix that was calculated from OTUs identified at the 5% level of dissimilarity. The centers of the ellipses correspond to the means of the clusters defined by the following groups: I, pre-fermented anaerobic digester (PF-AD) fed with large solids; II, PF-AD fed with all solids; and III, fermenter and manure samples (FERMAN). The radii of the ellipses were determined by 1.5 times the standard deviations from the mean and ellipses were oriented to encompass the maximum number of points in a group minus outliers.

Unless otherwise stated, similarity calculations at the 5% level of dissimilarity were used to delineate OTUs in the succeeding analysis. This level of dissimilarity, nominally grouping sequences at the genus level, was found to accurately predict the number of genera in an artificial mixture of short 16S rRNA gene sequences [15]. The similarities between the bacterial communities of the different bioreactors were compared by principal coordinates analysis (PCoA) on 21 bioreactor samples (Table 2) plus 3 manure samples and a compost sample that were included to characterize the baseline communities and as an out-group, respectively. As expected, the bacterial communities of fermenters, AD and compost were clearly separated by axis 1, which accounted for 21.4% of the variation in the data (Figure 1). Among the AD samples, the samples from the full-scale digesters (D1, D2) were clearly separated along axis 2 from the rest of the bench-scale AD reactors. There was no significant difference (p = 0.327) in the bacterial communities between manure and fermenter samples. The results also showed no clear difference between the overall bacterial communities of the SS-AD and PF-AD reactors. Substrate had a stronger effect on bacterial communities as compared to AD bioreactor type: as mentioned above, large-scale AD fed with a diverse source of manure (i.e., 11,000 cows, with each individual contributing some variation in fecal microbiota) could clearly be differentiated from other ADs. Sample T13, obtained from a PF-AD fed with the fine fraction of pre-fermented manure, could also be clearly differentiated from the rest of the AD samples. In Figure 1, group I represents a relatively tight cluster of points (signifying higher compositional similarity) centering around the mean coordinates of samples collected from PF-AD fed only with the coarse or large particle sized fraction of pre-fermented manure. Similarly, group II is centered around the mean coordinates of samples collected from PF-AD fed with all solids obtained by centrifugation from the manure fermenter. Compared to group I, samples fed with all solids tended to be more compositionally diverse and could not be clearly differentiated from SS-AD samples. Statistically, the difference between the two groups (PF-AD fed with either coarse or all solids) was significant although not highly so (p = 0.012). Overall, the result of PCoA suggest that bacterial communities of fermenter and manure (FERMAN) are statistically indistinguishable; and different manure feed types influences bacterial communities to a moderate (differences between fine/coarse/all fractions of manure) or high (diverse manure) degree.

### Analysis of Firmicutes and Bacteroidetes

**Figure 2:**
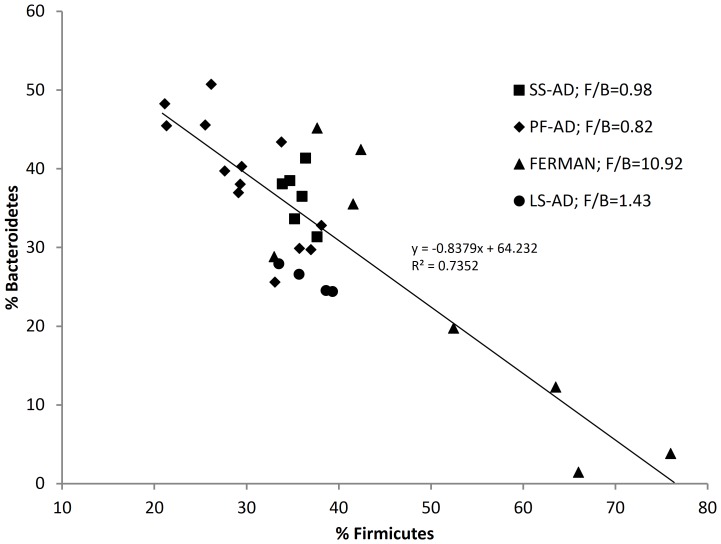
Relative abundances of *Bacteroidetes* vs. *Firmicutes* in FERMAN (fermenters and manure), SS-AD (single-stage anaerobic digesters), PF-AD (pre-fermented anaerobic digesters) and LS-AD (large-scale anaerobic digesters) as revealed by next-generation sequencing analysis. Target sample size was 96,000 sequences. F/B  =  average *Firmicutes* to *Bacteroidetes* ratio.

*Firmicutes* and *Bacteroidetes* constituted the two most abundant phyla in all AD samples analyzed, as well as most of the samples analyzed in the fermenters and manure samples. The exceptions were one manure and two fermenter samples – M3, F3, and F4, respectively, in which *Actinobacteria* were more dominant that *Bacteroidetes*. In general (for all bioreactors), the relative abundance of *Firmicutes* and *Bacteroidetes* averaged (*n* = 28) 70.0% (± *s.d.* 5.0) of the bacterial community. Plotting the relative abundances of *Bacteroidetes vs. Firmicutes* in all the bioreactor samples (plus three manure samples) revealed an overall negative relationship (Figure 2), which held true for three of the four groups, i.e., PF-AD, FERMAN and LS-AD, although the latter was represented by only four points, requiring caution in interpreting this relationship for LS-AD. Higher relative abundance of *Bacteroidetes*, ranging from 25.5% to 50.7% of bacteria, tended to be associated with PF-AD samples. The lower range of relative abundance of *Bacteroidetes* occurred in the fermenter (minimum of 3.8% of bacteria) and manure (minimum of 1.4% of bacteria) samples. Thus the highest relative abundance of *Firmicutes* also occurred in the fermenter (maximum of 75.9% of bacteria) and manure (maximum of 66.0% of bacteria) samples. Conventional single-stage AD and large-scale AD, both fed with raw manure and accomplishing primary fermentation and AD activities within a single vessel were associated with relatively narrower ranges of *Firmicutes* (33.9–38.1% and 33.5−38.6%, respectively) and *Bacteroidetes* (31.3–41.3% and 24.3–27.9%, respectively).

### Average and Core Microbiomes

**Figure 3:**
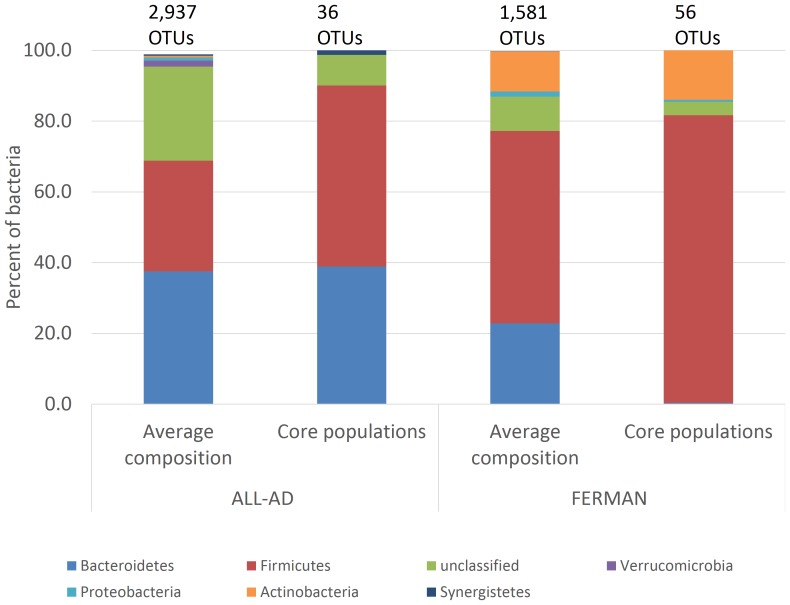
Average and core bacterial phyla associated with ALL-AD (all anaerobic digesters) and FERMAN (fermenters and manure). The average sample sizes for ALL-AD and FERMAN are 30,849 and 24,877 sequences, respectively. The phyla included in the figure comprise at least 99% of bacteria sequenced.

For the purpose of simplifying analysis, samples were grouped based on bioreactor source type or clustering obtained from PCoA. Manure and fermenter samples were combined into a single major group which we collectively refer to as FERMAN. The second major group consisted of all anaerobic digesters (ALL-AD  =  SS-AD + PF-AD + LS-AD). The average microbiome is determined mostly by differences in abundances of individual populations, while membership in the core microbiome connotes functional indispensability associated with a particular phylotype resulting in consistent presence throughout all the samples within a particular group. In the case of ALL-AD, the core microbiome consisted of only 36 OTUs, out of a total of 2,937 possible OTUs that can obtained by randomly sampling 30,849 sequences from a pool size of 163,200 ALL-AD sequences (Figure 3; *Firmicutes* and *Bacteroidetes* constituted on average 627 AND 723 OTUs, respectively). As expected, the majority of core OTUs were classified as *Firmicutes* and *Bacteroidetes* (14 OTUs each) while a single core OTU was classified as the *Synergistetes*. The latter finding is surprising considering that the *Synergistetes* was a rare phylum, present at only 0.42% of the bacteria in the average microbiome of ALL-AD. On the other hand, the *Verrucomicrobia*, which on average occurred at a higher frequency in ALL-AD, was not a core bacterial group in ALL-AD.

The core microbiome of the FERMAN was more diverse compared to ALL-AD and consisted of 56 OTUs, the majority of which (43 OTUs) were identified as *Firmicutes* (Figure 3). The dominance of the *Firmicutes* highlights its critical function in primary fermentation of manure. On average, *Bacteroidetes* were also a dominant phylum in FERMAN (347 *Bacteroidetes* OTUs on average) but only a single core *Bacteroidetes* OTU (consisting of 105 sequences) was consistently detected in all FERMAN samples; thus the *Bacteroidetes* was not dominant in the core microbiome of FERMAN. The exceptions to the dominance of *Bacteroidetes* occurred in one out of three manure samples and 2 out of 5 fermenter samples. In all these instances, the second most dominant phylum was the *Actinobacteria*. In summary, the core microbiome of fermenters and manure is dominated by the *Firmicutes*. Although not as abundant as the *Bacteroidetes*, more *Actinobacteria* sequences were consistently detected in all fermenter and manure samples, making this group the second most important core phylum in fermenters and manure, while the *Bacteroidetes* and *Proteobacteria* occupied less important niches in these systems.

### Key Bacteria Associated with Fermentation and Anaerobic Digestion

**Figure 4:**
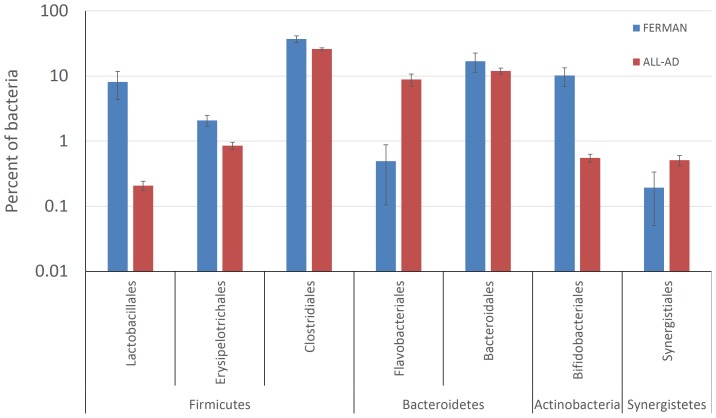
Key orders of bacteria belonging to *Firmicutes*, *Bacteroidetes*, *Actinobacteria*, and *Synergistetes* in FERMAN (fermenters and manure samples; *n* = 8) and ALL-AD (all anaerobic digester samples; *n* = 23). Error bars represent standard errors of the means.

Identifying the members of the core microbiome of primary fermenters and anaerobic digesters allowed us to analyze at greater sequencing depth: whereas beta-diversity analysis and classifications of average and core microbiomes relied on individual sample size of 9,600 sequences (pooled together totaling 240,000 sequences from all bioreactors plus manure and outgroup) – at this number of sequences, the minimum coverage value obtained was 0.81. A coverage value of 1.0 means all of the sequences were sampled more than once. At a larger sample size of 96,000, the minimum coverage value obtained was 0.93, making classifications, rarefactions, and diversity analysis more exhaustive at this larger sampling size. In the case of the *Bacteroidetes*, the two most frequently occurring orders were the *Bacteroidales* (12.4% and 17.8% of bacteria in ALL-AD and FERMAN, respectively) and *Flavobacteriales* (10.3% and 0.2% of bacteria in ALL-AD and FERMAN, respectively) (Figure S2). The distribution of these two bacteroidete orders in all the samples suggests that while *Bacteroidales* was consistently present in all bioreactors and manure, the order *Flavobacteriales* was more consistently associated with anaerobic digesters (Figure 4).

Within the *Firmicutes*, the order *Clostridiales* was dominant in all bioreactors and manure samples (Figure S3 and Figure 4). This is consistent with the known role of *Clostridia* in both fermentation and anaerobic digestion. Aside from the *Clostridia*, the orders *Erysipelotrichales* and *Lactobacillales* were detected at relative abundances reaching maxima of 4% and 30% of bacteria, respectively. Both *Erysipelotrichales* and *Lactobacillales* tended to be relatively more abundant in FERMAN – averaging (*n* = 8) 8.1% (±10.4) and 2.0% (±1.1) of bacteria, respectively compared to averages (*n* = 23) of less than 1% in ALL-AD for both groups.

Aside from the dominant phyla *Firmicutes* and *Bacteroidetes*, two minor core phyla associated with the FERMAN and ALL-AD were the *Actinobacteria* and *Synergistetes*, respectively. Within the *Actinobacteria*, the most dominant classified genus was *Bifidobacterium* (26–47% of actinobacterial sequences) while genus-level taxa within the *Synergistetes* could not be reliably classified for most sequences (11% of *Synergistetes* were classified as *Aminobacterium*).

## Discussion

Animal-to-animal variation in fecal bacterial community structure at the species level has been observed in cows [17], which probably reflects the process of speciation occurring at the individual cow level. Thus it is not surprising that genus and species level OTUs are most diverse in the large scale AD facility processing manure from thousands of cows. What is unclear at this point is the ecological significance or explanation for high phylum level richness in an ecosystem, such as that observed in fermenters.

*Bacteroidetes* and *Firmicutes* are dominant phyla in anaerobic systems, including anaerobic digesters, where they have been shown to be also stable when fed with manure and co-digested with different substrates such as casein, starch and cream [16], [18]. While there have been numerous microbial ecological studies on anaerobic digesters, to our knowledge, this study is the first to compare the bacterial communities in fermenters, SS-AD, PF-AD and LS-AD systems processing bovine manure. One study investigating the average and core microbiomes in several anaerobic digesters processing activated sludge found a core group consisting of *Chloroflexi*, *Betaproteobacteria*, *Bacteroidetes*, and *Synergistetes* [19]. Riviere et al. 2009 used a clone library-based approach and also identified the *Firmicutes* as the fourth most abundant group in activated sludge ADs. However, firmicute OTUs tended not to be shared across majority of the activated sludge ADs – and thus did not comprise a ‘core’ bacterial group. A similar study using next-generation 16S rRNA gene sequencing (pyrosequencing) identified similar average bacterial compositions in seven full-scale ADs processing activated sludge from municipal wastewater treatment plants (MWWTP) [20]. In our study, the core phylotypes identified were taxonomically less diverse compared to those found in WWTP ADs – specifically, *Chloroflexi*, *Proteobacteria*, *Spirochaetes*, and *Thermotoga* were relatively less abundant in AD processing dairy manure. On average, these groups were also present in dairy manure AD, but specific phylotypes were not shared across different reactor types. Differences in substrate, system configuration, scale, and analysis methods (e.g., clone library vs NGS) may account for the differences in the core microbiomes of different types of ADs. However, the consistent identification of *Synergistetes* across different studies [19, 20, 22, this study] suggests that this group of bacteria plays a consistent role in anaerobic digestion. While the role of *Synergistetes* in anaerobic systems remains largely unknown [21], there is experimental evidence to suggest that members of this group utilize acetate syntrophically with hydrogenotrophic methanogens [22]. Moreover, culture-based studies show that cultured representatives of this group are amino acid degraders with a limited repertoire of carbon sources (Godon et al. 2005 [21] and references within). This carbon source limitation dictates that while *Synergistetes* are very frequently present in anaerobic systems, they are present at low abundance – which is in agreement with our results.

*Bacteroidetes* and *Firmicutes* are also dominant in mammalian gut, with relative abundances that are determined by a number of host-related factors, such as obese or lean states [23] or age [24]. The intestinal microbiota may be one of many factors that can influence obesity – however the manner in which gut microbes do this is still unclear. Microbial contribution to obesity may be related to increased energy harvest which has been related to increased ratios of *Firmicutes* to *Bacteroidetes* during the obese state [25]. However, more recent findings suggest that although obesity and leanness are influenced by the gut microbial ecology, leanness is better described as the result of the interaction between microbial ecology and diet. Specifically, a higher relative abundance of *Bacteroidetes* combined with a high fiber diet are two conditions that promote leanness [4]. Moreover, energy harvest by a *Bacteroidetes*-dominated community from a high-fiber diet is greater compared to a *Firmicutes*-dominated community, which nevertheless promotes leanness, suggesting that energy harvest from substrate and obesity are not necessarily positively correlated. These findings are relevant to our study because we have consistently observed comparable methane yields from single- and two-stage digesters despite the latter being fed with manure depleted of a large fraction of its labile carbon through pre-fermentation. This can be explained by more efficient energy harvest from pre-fermented manure by a *Bacteroidetes*-dominated community comparable to energy harvest from a high-fiber diet by a *Bacteroidetes*-dominated gut community.

More efficient energy harvest may also be achieved by faster reaction kinetics, which is promoted in the primary stage fermenter that is operated at a much shorter solids retention time compared to the ADs. On average, primary stage fermenters exhibit microbiological traits linked to obesity: higher F/B ratios and a ‘diet’ that is less fibrous and more labile compare to that fed to PF-AD. That the *Bacteroidetes* is only a minor component of the core microbiome of manure fermenters does not suggest that this group is less functionally important in fermenters: our results suggest that hydrolysis and primary fermentation-related functions in *Bacteroidetes* are shared across *Bacteroidetes* phylotypes such as those identified in the average FERMAN microbiome (347 OTUs). Therefore, if majority of *Bacteroidetes* phylotypes carry out the same hydrolysis and primary fermentation-related functions, then any of these phylotypes can dominate different systems. Thus these functions are probably ancestral traits within the strictly anaerobic branch of the *Bacteroidetes* (*Bacteroidales*) which were consistently detected across all samples.

The *Bacteroidetes* are also known for two other attributes: this phylum is known to support frequent horizontal gene transfer events that allow the spread of novel metabolic capabilities such as the degradation of plant-derived fibers [26]. This phylum is also well known to include members specialized in biopolymer degradation [27]. Our more consistent detection of *Flavobacteriales* in the anaerobic digesters are consistent with this observation. Thus it makes great sense for this group of bacteria to be enhanced in PF-AD, which ultimately enables the system to produce comparable energy yields as SS-AD despite the re-directing of a substantial fraction of the manure's energy content to external uses (not AD) after pre-fermentation. Thus, the microbiology of animals and anaerobic digesters are intricately linked to their diet [28]. The populations of microbes that come to dominate both systems ultimately determine how much energy can be harvested from food.

While leanness is considered a healthier state, certain microbes associated with health also tended to be associated with primary fermenters. *Lactobacilli* sp. and *Bifidobacteria* sp. are typical components of probiotics and are recognized for their positive effects on human health especially in the prevention and treatment of intestinal disorders. Our observation that these groups of bacteria are mainly associated with FERMAN is evidence that certain attributes of the obese state, such as the presence of labile organic substances can promote health-associated bacteria. This may have implications on how to enhance the survival and growth of these bacteria when ingested as probiotics. In engineered systems, if the ultimate objective is to harvest more energy and value added products from manure, then our results argue in favor of operating separate “obese” and “lean” bioreactors (fermenters and PF-ADs). These facilitate the production of valuable co-products from the VFAs and comparable methane output as the less “lean” SS-AD that generates only methane and digestate. Overall, linking our findings with the science of gut microbiology demonstrates the possibilities of cross-pollination of knowledge in natural and engineered systems.

## Supporting Information

Figure S1 **Rarefaction curves of A) least OTU-rich manure sample and B) most OTU-rich LS-AD (large scale anaerobic digester) sample based on analysis of 96,000 sequences per sample.**
(TIF)

Figure S2 **Percentages of average and core orders of** ***Bacteroidetes*** **out of total bacteria associated with FERMAN (fermenter + manure) and ALL-AD (all anaerobic digesters).**
(TIF)

Figure S3 **Percentages of average and core orders of** ***Firmicutes*** **out of total bacteria associated with FERMAN (fermenter + manure) and ALL-AD (all anaerobic digesters).**
(TIF)
