# Supplementary figures and images for: Should We Build “Obese” or “Lean” Anaerobic Digesters?
Source: PLoS One. 2014 May 15;9(5):e97252. doi: 10.1371/journal.pone.0097252 (PMC4022501; doi:10.1371/journal.pone.0097252)

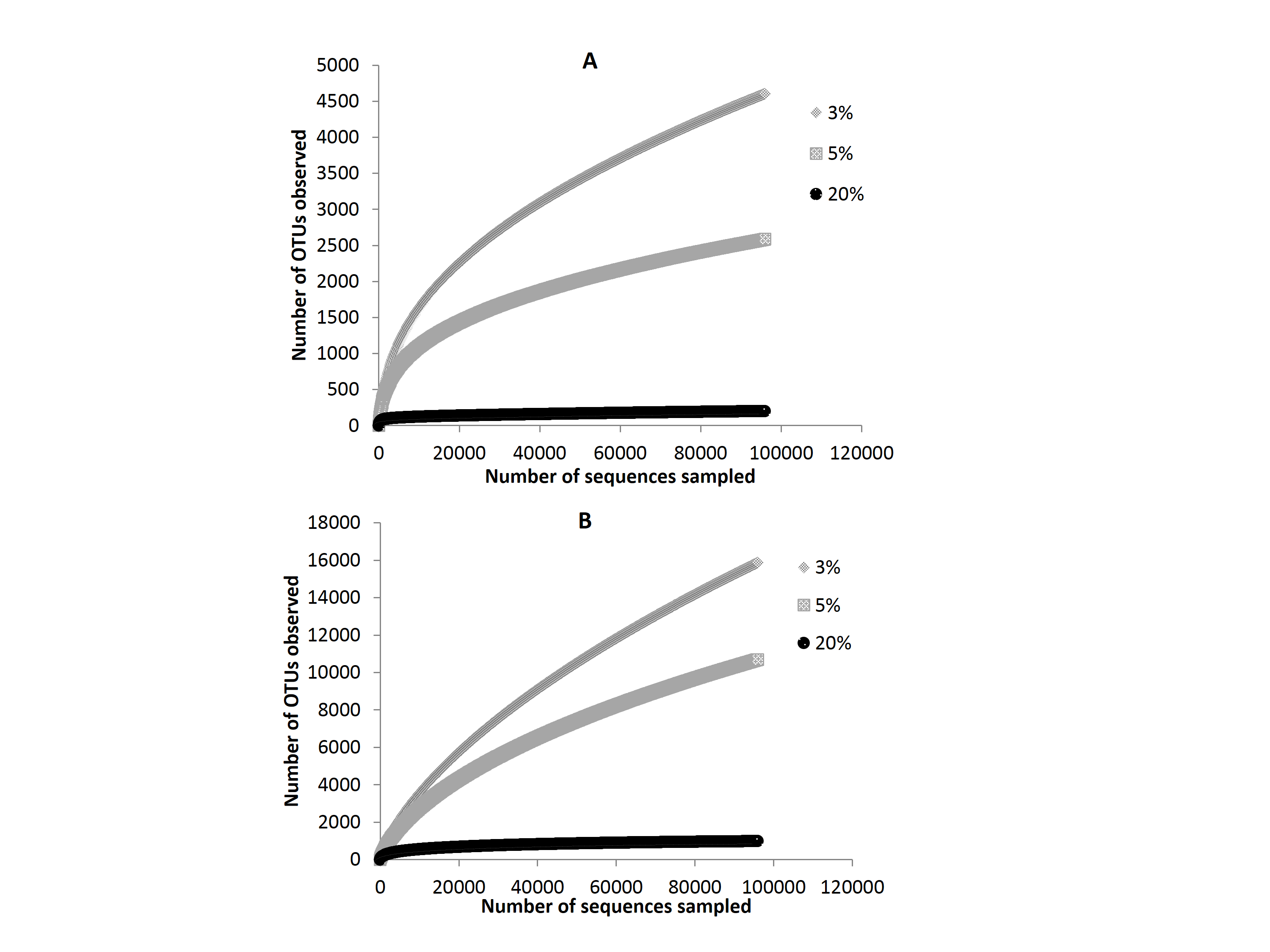

Supplement: Figure S1 — Rarefaction curves of A) least OTU-rich manure sample and B) most OTU-rich LS-AD (large scale anaerobic digester) sample based on analysis of 96,000 sequences per sample. (TIF) [file pone.0097252.s001.tif]

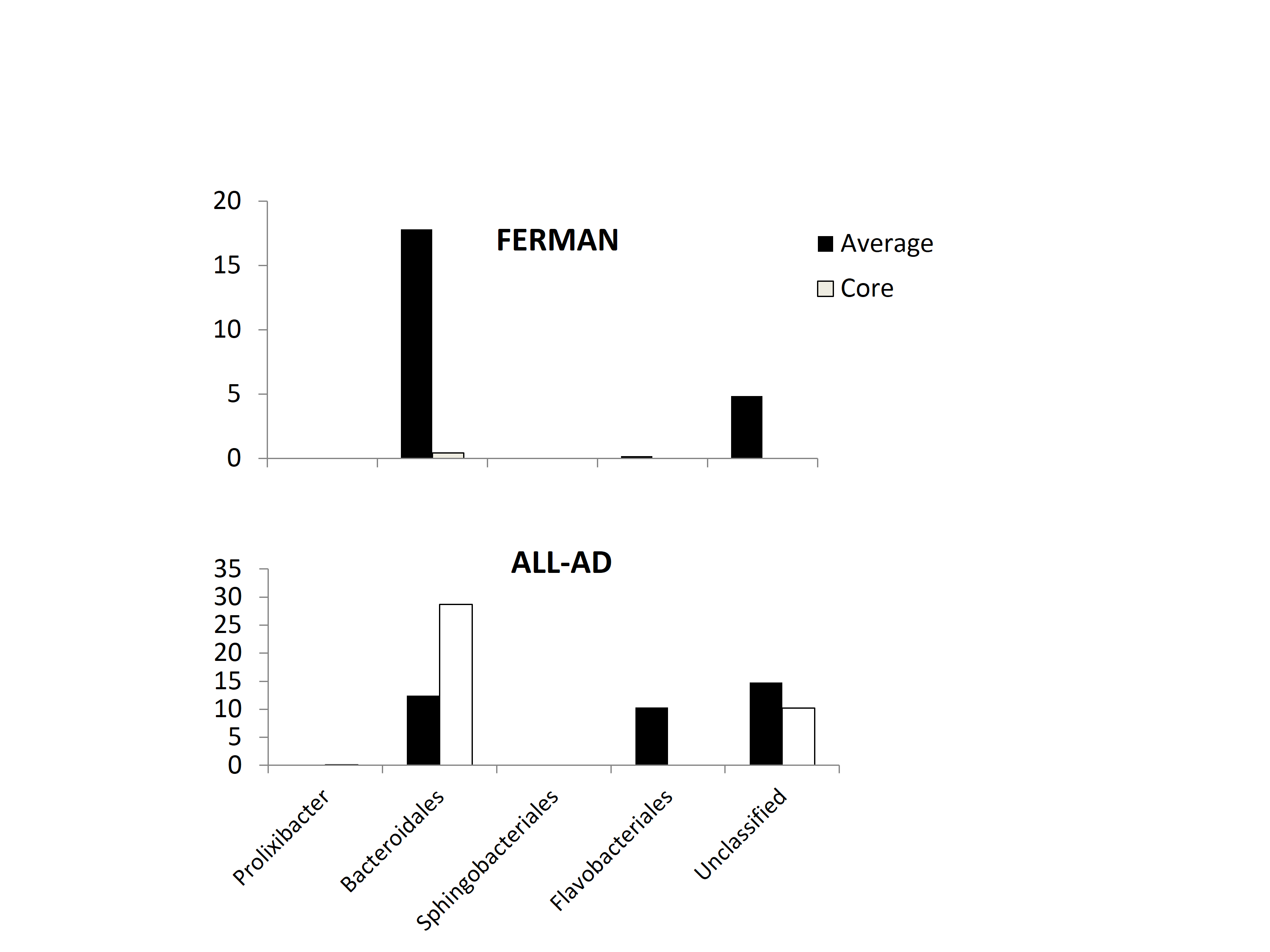

Supplement: Figure S2 — Percentages of average and core orders of Bacteroidetes out of total bacteria associated with FERMAN (fermenter + manure) and ALL-AD (all anaerobic digesters). (TIF) [file pone.0097252.s002.tif]

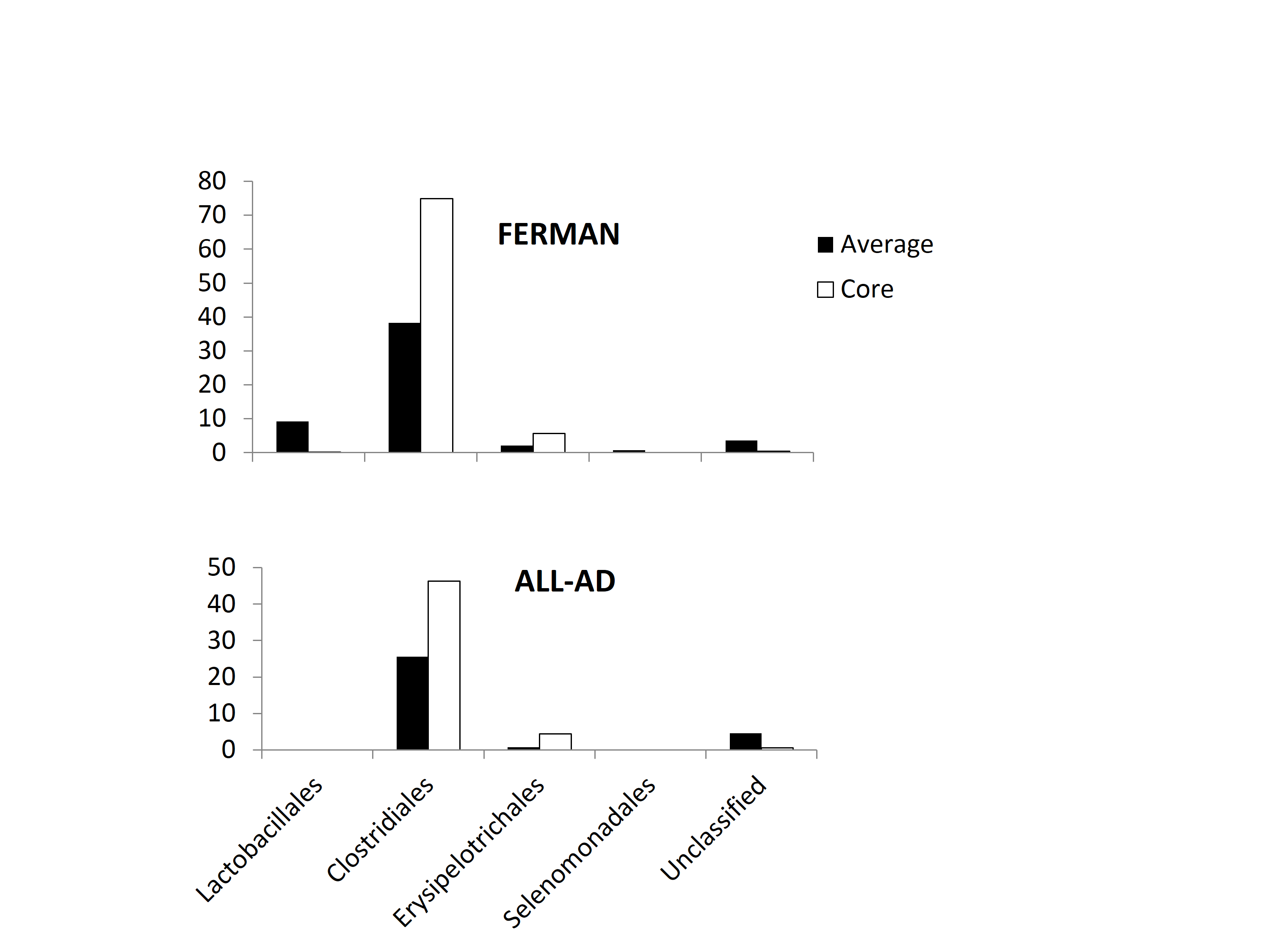

Supplement: Figure S3 — Percentages of average and core orders of Firmicutes out of total bacteria associated with FERMAN (fermenter + manure) and ALL-AD (all anaerobic digesters). (TIF) [file pone.0097252.s003.tif]
